# The acromegaly lipodystrophy

**DOI:** 10.3389/fendo.2022.933039

**Published:** 2022-09-13

**Authors:** Pamela U. Freda

**Affiliations:** Department of Medicine, Vagelos College of Physicians and Surgeons, Columbia University, New York, NY, United States

**Keywords:** acromegaly, growth hormone, lipodystrophy, adipose tissue, body composition, insulin resistance, ghrelin, AgRP

## Abstract

Growth hormone (GH) and insulin-like growth factor 1 (IGF-1) are essential to normal growth, metabolism, and body composition, but in acromegaly, excesses of these hormones strikingly alter them. In recent years, the use of modern methodologies to assess body composition in patients with acromegaly has revealed novel aspects of the acromegaly phenotype. In particular, acromegaly presents a unique pattern of body composition changes in the setting of insulin resistance that we propose herein to be considered an acromegaly-specific lipodystrophy. The lipodystrophy, initiated by a distinctive GH-driven adipose tissue dysregulation, features insulin resistance in the setting of reduced visceral adipose tissue (VAT) mass and intra-hepatic lipid (IHL) but with lipid redistribution, resulting in ectopic lipid deposition in muscle. With recovery of the lipodystrophy, adipose tissue mass, especially that of VAT and IHL, rises, but insulin resistance is lessened. Abnormalities of adipose tissue adipokines may play a role in the disordered adipose tissue metabolism and insulin resistance of the lipodystrophy. The orexigenic hormone ghrelin and peptide Agouti-related peptide may also be affected by active acromegaly as well as variably by acromegaly therapies, which may contribute to the lipodystrophy. Understanding the pathophysiology of the lipodystrophy and how acromegaly therapies differentially reverse its features may be important to optimizing the long-term outcome for patients with this disease. This perspective describes evidence in support of this acromegaly lipodystrophy model and its relevance to acromegaly pathophysiology and the treatment of patients with acromegaly.

## Introduction

**Figure 1:**
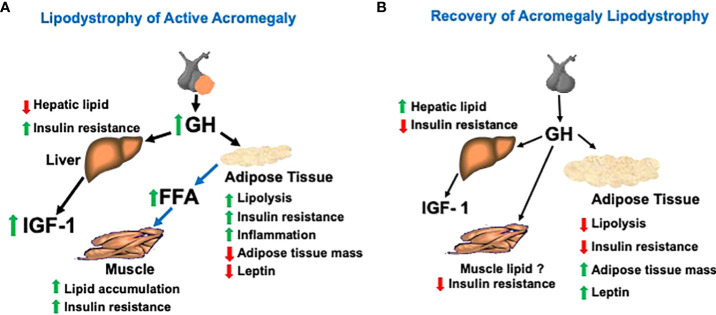
**(A)** Model of the acromegaly lipodystrophy that is present when the disease is active (i.e., elevated levels of GH and IGF-1). The lipodystrophy is initiated by a GH-induced accelerated lipolysis leading to insulin resistance, adipose tissue inflammation, and reduced adipose tissue mass, especially that of the VAT depot. Hepatic lipid is reduced, but hepatic insulin resistance occurs. Lipid is redistributed from VAT and SAT depots to ectopic deposition in muscle and may contribute to muscle insulin resistance. **(B)** Model of recovery of the lipodystrophy with biochemical remission after acromegaly treatment. After normalization of GH by surgery or medical therapy, adipose tissue lipolysis is reduced, permitting a re-accumulation of VAT and SAT lipid stores, a rise in intra-hepatic lipid and reduction in insulin resistance. Muscle lipid may not decrease due to the rise in total adipose tissue mass with acromegaly therapy despite improvement in insulin resistance.

GH and IGF-1 are vital to normal growth, metabolism, and body composition (1). In acromegaly, however, excesses of GH and IGF-1 markedly alter these processes. Changes in body composition and metabolic abnormalities are prominent features of the acromegaly phenotype and reflect, predominantly, direct actions of GH on peripheral tissues (2, 3). In particular, disordered glucose metabolism and insulin resistance (IR) are common at acromegaly diagnosis and often persist despite effective acromegaly treatment and reduce survival (4–11). In the general population, body fat quantity and distribution are important determinants of metabolic and cardiovascular risk (12–16). For example, increased visceral adipose tissue (VAT) mass associates with IR and type 2 diabetes (13, 17–19). However, this paradigm linking body composition pattern to metabolic abnormalities in the general population does not apply to acromegaly. Rather, acromegaly presents a unique constellation of these features that we propose to be considered an acromegaly-specific lipodystrophy (**Figure 1**). The lipodystrophy, initiated by a distinctive GH-driven AT dysregulation, features IR in the setting of reduced VAT and intra-hepatic lipid (IHL) but with lipid redistribution, resulting in ectopic lipid deposition in muscle (20, 21). How acromegaly therapies may differentially reverse the lipodystrophy’s features should be considered. This perspective describes evidence in support of this acromegaly lipodystrophy model and its relevance to acromegaly pathophysiology and treatment.

## Body composition in the lipodystrophy

The acromegaly lipodystrophy originates in AT under the influence of GH excess. GH modulates AT metabolism and is lipolytic (1, 22–26). Mechanisms for this predominate in VAT (19, 27–31). GH increases HSL, reduces LPL activity, activates the β-adrenergic 3 receptor and other lipolytic pathways, and inhibits lipogenesis (29, 32–44). GH may also impair adipocyte differentiation and adipogenesis (45–47). These effects act in concert to reduce AT mass in acromegaly (20, 48–50). Initial studies utilized a four-compartment body composition model that could not define specific depot changes (49, 51, 52), and although DXA finds reduced trunk fat (53, 54) and suggests VAT changes (55), techniques for VAT estimation by dual-energy X-ray absorptiometry (DXA) require validation in acromegaly. When directly quantified by whole-body MRI, both VAT and subcutaneous adipose tissue (SAT) mass were lower than predicted in active acromegaly (20). With surgical treatment, specific AT depot changes include increases in VAT and SAT, as quantified by CT (50) or whole-body MRI (20, 21) (**Table 1**). Pegvisomant therapy also increases intra-abdominal fat with a short-term treatment (56) and VAT and SAT with a long-term treatment (57) (**Table 1**). Limited data suggest increases in fat mass and decreases in lean mass (by DXA) with short-acting octreotide or Somatostatin receptor ligands (SRL) therapy (58, 59). Body composition changes in acromegaly relate to disease activity, including IGF-1 levels (54, 59) and rise in VAT mass after surgery correlates with the decrease in GH (21). Interestingly, AT mass changes, especially those of VAT, are greater in men than women with acromegaly (20, 53, 60). Mechanisms for this are incompletely understood, but known gender differences in body composition (61) and greater sensitivity of VAT to GH in men (62) may contribute to these differences. VAT may rise to above expected in men (21), but in one study, VAT lowering followed an early post-operative rise (60) and another suggested that gender differences attenuate in the long-term (59). Further investigation into the time course, patterns, and long-term outcome of AT mass changes after acromegaly therapy in men vs. women is warranted. Given the reduced AT mass in acromegaly, dysfunctional AT appears to be a greater determinant than absolute AT mass to the metabolic abnormalities. The contributions of the former and its changes with acromegaly treatment are incompletely understood yet highly relevant to optimizing therapy.

**Table 1:** Body composition changes in patients with acromegaly treated with surgery (top) or pegvisomant therapy (bottom).

	# Patients (Men/Women)	Age (Years) (Median, Range)	Prior Therapy	VAT Mass	SAT Mass	SM Mass	Intra-Hepatic Lipid: % Water Signal (MRS)
**A: Surgical therapy**							
**Pre-surgery**	**10/13**	**44 (18–69)**		**Percent change from baseline values**			Men: 0.0137 ± 0.02; Women: 0.0098 ± 0.006; Men and women: 0.012 ± 0.02
**Post-operative**							
**6 months**							
** **Men				86.4 ± 71 (*P* = 0.03)	17.6 ± 7.4 (*P* = 0.015)	−5.56 ± 6.8	
** **Women				7.98 ± 19 (*P* = 0.049)	5.5 ± 5.7 (*P* = 0.03)	1.9 ± 16.8	
**1 year**							
** **Men				112.8 ± 93 (*P* = 0.006)	19.9 ± 15 (*P* = 0.01)	−7.6 ± 6.8 (*P* = 0.01)	0.034 ± 0.06
** **Women				29.7± 27 (*P* = 0.03)	7.7 ± 14.5	−0.975 ± 8.3	0.016 ± 0.01
** **Men and women							0.026 ± 0.04 (*P* = 0.03)
**2 years**							
** **Men				161.7 ± 76 (*P* = 0.03)	27 ± 12.6 (*P* = 0.03)	−9.53 ± 2.4 (*P* = 0.01)	
** **Women				46 ± 42 (*P* = 0.03)	19 ± 16.9	−5.27 ± 6.6	
**B: Pegvisomant therapy**							
**Pre-pegvisomant**	**12/4**	**48 (19–62)**	**S (16), C(11), BC (4), P (1),** **SA (13), RT (8)**				0.022 ± 0.01
On-pegvisomant*							
** 1–2 years**	16			60.1 ± 56.1(*P* = 0.003)	3.9 ± 11.9	−0.583 ± 6.6	0.043 ± .03 (*P* = 0.04)
** 3–4 years**	6			99.3 ± 52.1 (*P* = 0.002)	23.7 ± 21.2 (*P* = 0.04)	0.356 ± 6.8	
** 5–6 years**	6			88.5 ± 50.9 (*P* = 0.007)	17 ± 17.7 (*P* = 0.06)	−2.942 ± 6.9	
** >8 years**	4			138.7 ± 7 (*P* = 0.045)	19.1 ± 21.7	−1.087 ± 10.6	

Top: Total body MRI measured percent changes in VAT (visceral adipose tissue), SAT (subcutaneous adipose tissue), and SM (skeletal muscle) masses from pre-operative values to those at 6 months, 1 year, and 2 years after surgery in men and women, separately. Mean ^1^HMRS measured intra-hepatic lipid (IHL) pre-operatively and 1-year post-operatively in men, women, and men and women combined. Bottom: Changes in VAT, SAT, and SM mass from pre-pegvisomant baseline to 1–2 years, 3–4 years, 5–6 years, and ≥ 8 years of pegvisomant therapy in men and women combined. Mean ^1^HMRS IHL in acromegaly men and women combined, pre-pegvisomant and after 1–2 years on pegvisomant.

Adapted from the author’s work in references (21, 57).

Data are mean ± SD, unless otherwise noted.

P-values represent significance of change from pre-therapy (baseline) to each post-operative or on pegvisomant follow-up time point.

Types of prior therapy: S, transsphenoidal surgery; C, cabergoline; BC, bromocriptine; P, pergolide; SA, long-acting somatostatin analog; RT, radiotherapy (number of patients).

*Men and women combined.

GH has important effects on protein metabolism that favor anabolism in muscle (63, 64), and some evidence for this in acromegaly exists (65, 66). Recent advances in body composition testing methodologies have also enabled the assessment of skeletal muscle (SM) mass in acromegaly. In patients with active acromegaly, SM mass did not differ from predicted (by whole-body MRI) (21, 67) but was higher [by Bioelectrical Impedance Analysis (BIA)] in pre-operative patients (60). Whereas earlier studies reported no change in body cell mass with acromegaly treatment (68), subsequent studies found decreases in SM volume (by CT) (50) and in SM mass by BIA (60) and by MRI (in men) (20) after surgery (**Table 1**). SM mass did not change with the long-term pegvisomant therapy (57) (**Table 1**). In cross-sectional studies, lean tissue mass (by DXA) was increased in active acromegaly compared with that in remission acromegaly (53, 54, 69). Importantly, however, DXA lean tissue estimates are not a surrogate for SM in acromegaly, as they include measures of soft tissues (51, 70) that are influenced by the increased tissue hydration of acromegaly (48, 49). In fact, changes in DXA lean tissue with acromegaly treatment are accounted for by those of the non-SM components that make up lean tissue (57, 67). Genders differ in the interaction of acromegaly disease activity with lean or SM changes (54), which are greater in men (53). SM mass is likely influenced by gonadal steroid changes in men with acromegaly (66, 71). Overall, reductions in SM mass with acromegaly therapy are small, and reports on the effect of acromegaly and its treatment on SM function vary (72–75), but the impact on SM metabolism and other outcomes requires further study.

MRI and proton magnetic resonance spectroscopy (^1^HMRS) imaging use has also enabled visualization of muscle lipid content in acromegaly. MRI revealed that inter-muscular AT (IMAT), AT located between muscle groups and beneath the fascia (76–78), is increased in active acromegaly. This is an important feature of the lipodystrophy (20). GH-induced AT lipolysis may lead to lipid movement from VAT and SAT depots to muscle where it is deposited ectopically. Free fatty acid (FFA) flux and uptake in muscle are increased in acromegaly and with GH use (32, 79–82), and supraphysiologic GH increased intra-myocellular lipid (IMCL) on SM biopsy (83). In other settings, FFA rise is associated with an increase in IMCL (84, 85) and IMAT (86–89) and with IR (90, 91). Anti-lipolysis along with GH administration reduces its effect on muscle IR (32, 92), supporting a role for muscle lipid in IR in acromegaly. IMAT may relate to IR in acromegaly (20), but IMAT was lowered only in women after surgery and not lowered with pegvisomant therapy despite improved IR (21, 57). Increases in total AT, a major determinant of IMAT, may obscure IMAT changes with acromegaly therapy. IMCL did not change with surgery (21, 93) or in pegvisomant-treated acromegaly patients vs. controls (94) but did decrease with the addition of pegvisomant to SRL therapy (95) and correlated inversely with insulin sensitivity in a combined acromegaly and control cohort (94). Further study is needed to understand the effects of acromegaly and its therapy on muscle lipid and its relationship to IR in acromegaly.

Another key component of the acromegaly lipodystrophy revealed by ^1^HMRS is that IHL is low in active acromegaly in the setting of IR (21, 93). This appears to contrast with the positive correlation between IHL and IR in other populations (96). Other data support an influence of GH on liver fat: mice with impaired GH signaling or GH receptor deletion have increased liver fat and steatosis (97–99), and, in case reports, liver fat was increased in patients with growth hormone deficiency (GHD) (100–102). In small cross-sectional studies, IHL was up to three-fold higher in treated acromegaly vs. controls (94, 103). IHL rises with surgical therapy (21, 93, 104), when pegvisomant is added to SRL therapy (95) and with long-term pegvisomant monotherapy (57) (**Table 1**). On pegvisomant, IHL was similar to controls, suggesting that it returns to the expected levels on therapy (57). It seems that hepatic lipid accumulation cannot be implicated in hepatic IR in acromegaly (96), but whether other characteristics of IHL can, for example, altered proportions of certain lipid species (105) requires further study.

Amount and distribution of AT are important determinants of IR in acromegaly (54, 106). In other lipodystrophies, inability to store lipid in SAT is thought to promote ectopic lipid deposition and IR, and, conversely, lipid storage in SAT may be protective for ectopic lipid deposition, IR and diabetes (12, 14, 107, 108). Potentially, in the acromegaly lipodystrophy, inability to store lipid in SAT contributes to IR, and SAT re-expansion with its recovery is important to its resolution. We found that lowering of homeostatic model assessment (HOMA) score correlated with SAT and VAT increases after surgical treatment (21), but others found less rise in trunk fat after surgery to be associated with greater improvement in HOMA score (55). These relationships require further study.

## Insulin resistance in the lipodystrophy

IR, a central feature of acromegaly’s metabolic abnormalities, is thought to be due primarily to GH’s insulin-antagonistic and lipolytic effects (63, 79, 109–112). IGF-1’s insulin agonism may partially offset those of GH, but circulating IGF-1 has a little role in regulating glucose homeostasis in acromegaly (10). Both hepatic and peripheral IR occur in acromegaly (79, 109, 110, 112, 113). Mechanisms for this are incompletely understood but may include impaired insulin signaling and substrate competition (10, 114–117). In other settings, IR relates strongly to increased FFAs from lipolysis by a number of proposed mechanisms (41, 63, 90, 116–134). GH increases FFA flux into muscle, which may increase muscle lipid and IR as described above (32, 79–81, 83–85, 92). Interestingly, although GH administration acutely increases FFA levels (43) and leads to increased muscle IR (135, 136), circulating FFA levels are not consistently elevated in acromegaly (110, 137, 138) but acromegaly treatment lowers them along with IR (139).

The importance of IR in AT has recently been emphasized (106, 140) and is a central feature of the acromegaly lipodystrophy. Dysfunctional AT is a major contributor to systemic IR in other populations (141). GH has complex effects that impair insulin action in AT and decrease its uptake and utilization of glucose (44, 114, 142–144). In acromegaly, accelerated lipolysis is the likely inciting precipitant of AT IR (140). In other models, disordered lipid metabolism in AT, particularly lipolysis and FFA release, may signal to induce inflammation in AT, which, in turn, promotes IR (145–154). In mice, GH excess is associated with immune and inflammatory changes in AT (155). *In vitro*, GH induced pro-inflammatory cytokines in pre-adipocytes yet suppressed them in AT macrophages (ATMs) (156). Because ATMs buffer lipid increases (145, 146, 148, 149) and the lack of functional GHRs in macrophages is associated with inflammatory ATM migration and IR in AT (157), acromegaly’s effects on immune components of AT could contribute to AT IR.

GH-induced changes in adipokines may also contribute to the development of IR in acromegaly. GH reduces leptin gene expression in VAT (158), and in acromegaly, circulating leptin levels are low (159–162) and rise with surgical (159) or pegvisomant (57, 163) therapy. Whether leptin changes are explained by or independent of those in fat mass is unclear (57, 164–167). Leptin deficiency contributes to IR and abnormal metabolism in other settings (168–170): leptin therapy corrects hyperglycemia in diabetic mice (171, 172) and lipodystrophy patients (170, 173, 174). However, changes in leptin and IR with acromegaly treatment do not consistently correlate (21), and whether low leptin contributes to IR in acromegaly and its rise to IR improvement is unknown. Circulating levels of visfatin (175) were increased in acromegaly in some (106, 160, 176) but not other studies (177) and correlated with those of IGF-1 (176), variably with IR (176, 178) and inversely with percentage body fat (160). GH increases visfatin expression in mature human adipocytes, supporting a pathogenic role in AT inflammation in acromegaly (160). Data conflict with regard to adiponectin levels in acromegaly, these were reduced in some (106, 177, 179) but not other (160, 161) studies. Some *in vitro* data suggest that GH may reduce adiponectin gene transcription (180). GH modulates 11B-HSD1 in AT and in acromegaly (34, 181–183), which could also play a role the lipodystrophy and its recovery.

## Ghrelin in the lipodystrophy

The effect of acromegaly on ghrelin is also relevant to the lipodystrophy. Ghrelin is an orexigenic hormone that is importantly linked to appetite and body composition (184–186) and to the GH–IGF-1 axis as a stimulator of pituitary GH secretion (187). Evidence supports that GH excess suppresses ghrelin: Ghrelin levels are lowered in active acromegaly (159, 188, 189) and rise with surgical treatment (159, 188). Most rodent data (190–192) and the finding that high-dose GH suppresses ghrelin (193) are consistent with this. In acromegaly, ghrelin levels correlated inversely with insulin levels and HOMA score in most studies (159, 188, 189), and ghrelin rise was inversely related to the decrease in these with surgery (159, 188). Other data support that hyperinsulinemia suppresses ghrelin (194–197). Interestingly, in one study, rise in ghrelin correlated with increase in body fat with surgical remission (159). These may be related. In rodents, ghrelin induces lipogenesis, reduces fat utilization, and promotes weight gain (198–200), and in humans, ghrelin increased food intake (201) and promoted weight gain. Ghrelin and body fat also relate inversely during GH therapy (202). Transition from the state of increased lipolysis and energy expenditure (EE) before to that favoring lipogenesis and decreased EE after surgery (48, 110) could, in part, reflect ghrelin effects. A GH-overexpressing rodent model featured increased EE and resisted diet-induced obesity (203). Although a rise in ghrelin with increasing fat mass may seem paradoxical, ghrelin is dysregulated in active acromegaly, but after surgical remission, this may be reset to the expected negative correlations between ghrelin levels and BMI. By contrast, somatostatin analogs (SRLs) suppress ghrelin levels (188, 204, 205). Potentially, ghrelin suppression protects from weight gain similarly to the resistance to diet-induced obesity and increased EE of mice lacking ghrelin receptors (206). However, it is unknown whether SRL therapy is associated with less gain in central adiposity than other acromegaly therapies, but if shown, this would be important to consider in choosing an acromegaly therapy. SRL suppression of other gut and pancreatic hormones, which may impair glucose tolerance despite biochemical control, may also contribute to their effect on the acromegaly lipodystrophy and its metabolic consequences (207).

## Agouti-related peptide in the lipodystrophy

GH has well-known direct effects on metabolism in peripheral tissues (208), but recent data show that GH also acts in brain to control energy metabolism (209, 210). Systemic GH administration in mice was shown to activate Agouti-related peptide (AgRP) neurons to produce orexigenic responses, and GH receptor antagonism with pegvisomant attenuated them (209). Transgenic central nervous system (CNS) GH overexpression increased hypothalamic AgRP and Neuropeptide Y(NPY) expression and food intake (211). These data suggest that GH stimulation of AgRP may be a mechanism by which GH restores energy homeostasis during nutrient deprivation (209). Because hypothalamic AgRP promotes food intake and weight gain (212) and plasma AgRP levels rise with caloric restriction in humans (213), AgRP may mediate the metabolic effects of GH’s rise in physiologic settings that need nutritional intake (214). AgRP-Growth Hormone Releasing Hormone (GHRH) neuron interaction may also couple nutritional status with growth (82). In humans, plasma AgRP levels increase after acute and chronic caloric restriction and relate to nutritional state in the pattern expected for hypothalamic AgRP (213, 215–217). We recently found that the plasma AgRP levels are higher in active acromegaly than in matched healthy subjects and are lower after surgery that reduced GH/IGF-1 or pegvisomant that lowered IGF-1 levels (218), suggesting that GH/IGF-1 and AgRP are positively related. AgRP and proopiomelanocortin (POMC) neurons also integrate CNS pathways that modulate glucose utilization and production (219–224). In healthy humans, the plasma AgRP levels relate to insulin levels and HOMA score (213, 215, 216), and, in mice, evidence supports an effect of AgRP neuron activation to impair glucose metabolism (220, 225–231). These data suggest a possible role for GH excess, acting centrally on AgRP, in the metabolic abnormalities of acromegaly. Although, in acromegaly, the peripheral actions of chronic GH excess to reduce AT stores predominate clinically over AgRP’s central mechanisms that, conversely, promote adiposity and fatty liver (230, 231), the rise in AgRP could prevent even more fat loss in this setting. A potential role for the GH-AgRP axis in the acromegaly lipodystrophy warrants further study.

## Conclusions

The acromegaly-specific lipodystrophy features IR in the setting of a unique body composition pattern of reduced VAT and IHL and of impaired lipid storage in SAT, resulting in ectopic lipid deposition in muscle (20). The lipodystrophy results from a complex interplay of direct effects of GH on AT, primarily driven by accelerated lipolysis and the resultant promotion of IR. The effects on adipokines, ghrelin, and AgRP may also be important to producing IR in the face of low AT storage, as well as promoting AT mass regain along with reductions in IR during the lipodystrophy recovery. Increases in body fat with recovery of the lipodystrophy are nearly balanced by reduction in non-SM lean tissues such that only small increases in body weight occur (21, 159). Acromegaly therapies act by different mechanisms and at different targets along the GH–IGF-1 axis and may not impact all aspects of the lipodystrophy similarly. Gender differences in the lipodystrophy and its recovery require further study. Further investigation on the mechanisms of IR in AT, how AT distribution changes relate to IR, and the role of muscle lipid in IR in active acromegaly and during its treatment is warranted. Pegvisomant seems to reverse the acromegaly lipodystrophy pattern similarly to surgical therapy, but modern body composition methods have not yet been used to assess how it changes with SLR therapy. Interestingly, biochemical control of acromegaly reduces cardiovascular (CV) disease despite the post-treatment rise in VAT mass that, in the general population, is linked to CV risk. Whether VAT/IHL rise with acromegaly treatment persists and whether overtreatment could lead them to become above normal and potentially effect cardiovascular or diabetes risk require further study. Increases in fat mass may impact negatively on patients’ body image (232) and worsen quality of life (233) despite effective acromegaly treatment, and the development of mechanistic-directed therapies aimed at mitigating this during acromegaly therapy should be considered. Mechanisms for the acromegaly lipodystrophy are not fully elucidated, and understanding its pathophysiology and how therapies differentially impact its recovery is important to optimizing the long-term outcome for patients with this disease.

## Data availability statement

The original contributions presented in the study are included in the article/supplementary material. Further inquiries can be directed to the corresponding author.

## Ethics statement

The studies involving human participants were reviewed and approved by Institutional Review Board, Columbia University Irving Medical Center. The patients/participants provided their written informed consent to participate in this study.

## Author contributions

The author confirms being the sole contributor of this work and has approved it for publication.

## Funding

This work was funded by NIH grant DK110771.

## Conflict of interest

The author declares that the research was conducted in the absence of any commercial or financial relationships that could be construed as a potential conflict of interest.

## Publisher’s note

All claims expressed in this article are solely those of the authors and do not necessarily represent those of their affiliated organizations, or those of the publisher, the editors and the reviewers. Any product that may be evaluated in this article, or claim that may be made by its manufacturer, is not guaranteed or endorsed by the publisher.
